# Functionalization of stable fluorescent nanodiamonds towards reliable detection of biomarkers for Alzheimer’s disease

**DOI:** 10.1186/s12951-018-0385-7

**Published:** 2018-08-10

**Authors:** Francisco Morales-Zavala, Nathalie Casanova-Morales, Raúl B. Gonzalez, América Chandía-Cristi, Lisbell D. Estrada, Ignacio Alvizú, Victor Waselowski, Fanny Guzman, Simón Guerrero, Marisol Oyarzún-Olave, Cristian Rebolledo, Enrique Rodriguez, Julien Armijo, Heman Bhuyan, Mario Favre, Alejandra R. Alvarez, Marcelo J. Kogan, Jerónimo R. Maze

**Affiliations:** 1 Department of Pharmacological and Toxicological Chemistry, Facultad de Ciencias Químicas y Farmacéuticas, Universidad de Chile, Santiago, Chile; 2 Advanced Center for Chronic Diseases (ACCDiS), Santiago, Chile; 3 Institute of Physics, Pontificia Universidad Católica de Chile, Santiago, 7820436 Chile; 4 Department of Cellular & Molecular Biology, Pontificia Universidad Católica de Chile, Santiago, Chile; 5 CIBQA, Universidad Bernardo O’Higgins, Santiago, Chile; 6 Núcleo de Biotecnología Curauma, Pontificia Universidad Católica de Valparaíso, Valparaíso, Chile; 7 Center for Nanoscale Technology and Advanced Materials, Pontificia Universidad Catolica de Chile, Santiago, Chile; 8 CARE-Chile-UC, Pontificia Universidad Católica de Chile, Santiago, Chile; 9 Centro de Investigación en Recursos Naturales y Sustentabilidad, Universidad Bernardo O’Higgins, Santiago, Chile

**Keywords:** Fluorescent markers, Nanodiamonds, Peptide R7-CLPFFD, Alzheimer’s disease, Amyloid beta peptide

## Abstract

**Background:**

Stable and non-toxic fluorescent markers are gaining attention in molecular diagnostics as powerful tools for enabling long and reliable biological studies. Such markers should not only have a long half-life under several assay conditions showing no photo bleaching or blinking but also, they must allow for their conjugation or functionalization as a crucial step for numerous applications such as cellular tracking, biomarker detection and drug delivery.

**Results:**

We report the functionalization of stable fluorescent markers based on nanodiamonds (NDs) with a bifunctional peptide. This peptide is made of a cell penetrating peptide and a six amino acids long β-sheet breaker peptide that is able to recognize amyloid β (Aβ) aggregates, a biomarker for the Alzheimer disease. Our results indicate that functionalized NDs (fNDs) are not cytotoxic and can be internalized by the cells. The fNDs allow ultrasensitive detection (at picomolar concentrations of NDs) of in vitro amyloid fibrils and amyloid aggregates in AD mice brains.

**Conclusions:**

The fluorescence of functionalized NDs is more stable than that of fluorescent markers commonly used to stain Aβ aggregates such as Thioflavin T. These results pave the way for performing ultrasensitive and reliable detection of Aβ aggregates involved in the pathogenesis of the Alzheimer disease.

**Electronic supplementary material:**

The online version of this article (10.1186/s12951-018-0385-7) contains supplementary material, which is available to authorized users.

## Background

Since the discovery of Green Fluorescent Protein (GFP) in 1962 [1], fluorescent markers have revolutionized the field of bioimaging. These markers have endowed different biomolecules and cells the ability to fluoresce and therefore to become detectable by conventional optical microscopes [2]. Fluorescent markers have made possible the localization of organelles otherwise invisible, the tracking of biomolecules inside the cell, the study of chemical reactions of several biological processes [3, 4], and the analysis of molecular interactions using fluorescence resonance energy transfer (FRET) [5–7], to name just a few examples. Although all these new applications and techniques have greatly impacted the fields of biology and chemistry the use and development of fluorescent markers are still facing great challenges. Several fluorescent markers based on molecules and proteins present photo bleaching and blinking [8] decreasing the reliability of the studies in which they are used. Although the development of more stable fluorescent markers such as quantum dots [9–12] (QD) has shown great progress during the last 5 years, many semiconductor-based color markers are still toxic to the cell. Many color markers have short lifetimes compared to the time scale required for biological studies in order to reach dependable conclusions [13–15]. Therefore, stable fluorescent markers are essential for long experiments.

On the other hand, the ability of a marker to fluoresce is not enough. Fluorescent markers should be linked or conjugated in order to tag a specific molecule, organelle or to study a specific process. For example, several nanoparticles (NPs) have been designed to be used in specific biomedical and nanotechnological applications [16] by directing them to the correct place inside the body by either passive or active targeting [17]. Passive targeting is based on the inherent properties of nanoparticles or tissue abnormalities that allow them to accumulate at specific locations as in the case of Enhanced Permeability and Retention (EPR) effect present in some tumors [18]. Active targeting is based on the functionalization of the nanoparticle surface with signal molecules. In the past 30 years, several targeted nanoparticles functionalized with different ligands such as small molecules, polysaccharides, peptides, proteins, or even antibodies have been developed for therapeutic and diagnostic applications [19]. Nanoparticles have been used in preclinical studies to attack tumors [20], enhance drug delivery [21], and eliminate amyloid aggregates related to Alzheimer’s disease (AD) [22], just to name few examples. Therefore, the functionalization of nanoparticles by surface treatment or specific molecular conjugation is a key issue.

In this work, we present the functionalization of nanodiamonds (NDs)—which host stable fluorescent color centers—with a functional peptide in order to detect the extracellular accumulation of amyloid β (Aβ) peptide, which is believed to underlie the neuronal damage and cognitive decline in AD.

AD, the most common form of dementia in elderly people, is a progressive neurodegenerative disorder characterized by cognition and memory impairments. One of the main neuropathological features of the AD brain is the presence of senile plaques composed of aggregated Aβ peptide [23–26]. Therefore, several types of nanoparticles have been proposed for detecting this peptide [27, 28], which is a very specific biomarker for AD. In order to detect the Aβ peptide we used NDs. These nanoparticles have unconditionally stable fluorescence, even after several months under continuous wave excitation; they are biologically and chemically inert; and they can be used as sensors with sub-wavelength resolution [29]. We have functionalized the NDs surface (fND) with the bi-functional peptide R7-CLPFFD, composed of the CLPFFD peptide and a RRRRRRR (R7) peptide. The CLPFFD peptide is a β-sheet breaker that recognizes toxic extracellular aggregates of amyloid Aβ peptide present in the brain of AD patients [30]. Previously, this peptide had been attached to gold nanoparticles and showed selectivity to Aβ aggregates [31–33]. The R7 section is a cell penetrating peptide (CPP) that enhances the cellular uptake of its cargo [34]. For example, oligoarginines have been used to improve the delivery of drugs such as insulin when administered intranasaly [35–37]. These CPP are useful for the treatment of diseases that require the cross of different kinds of cellular barriers, such as the brain–blood barrier (BBB) in AD [38, 39].

Here we show that fNDs can be internalized in fibroblast cells and in bend.3 cells, a brain vascular endothelial cell line commonly used in in vitro models to test the transport through the BBB. At the same time, we show that fNDs bind to Aβ fibrils. Therefore, fNDs can be used for indirect detection of extracellular Aβ aggregates. Finally, we show that the fluorescence stability of fNDs is superior to that of common color markers used to stain Aβ such as Thioflavin T and FITC. Therefore, these results might enable longer and more reliable studies of Aβ aggregates.

## Results and discussion

### Properties of fluorescent nanodiamonds

**Fig. 1:**
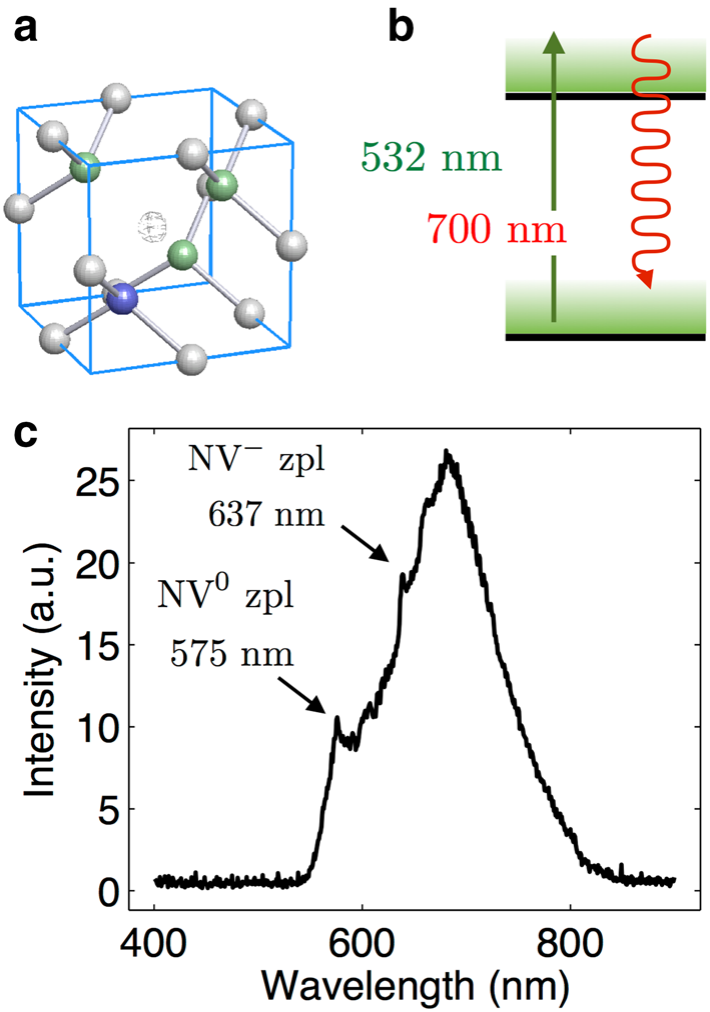
Emission properties of NV color centers in diamond. **a** Atomic configuration of NV color defect in diamond. One nitrogen (blue) and three carbons (green) are adjacent to a vacancy site. The NV center can exist in two charge configurations, the neutrally charge NV0 center and the negatively charged NV− center. **b** Two level model of the electronic transitions of the NV− color center. **c** Emission spectrum of nanodiamonds. The spectrum shows a zero phonon line at 575 nm for the NV0 center and at 637 nm for the NV− center. Both centers show a broad phonon side band

Diamond based fluorescent markers use color defect centers as their active emitting part. Defects in the crystalline structure of diamond can lead to localized electronic states within the diamond band gap, which is of the order of 5.6 eV [40]. Due to this large band gap, non-defective crystalline diamond exhibits fluorescence only if a very short wavelength laser is used. In addition, when a defect is created, not only its ground state but also its first optically exited state might exist within this band gap. Therefore, several different and stable optical defects can be hosted in the diamond matrix [41, 42]. A common color center is the nitrogen-vacancy (NV) center whose atomic structure is shown in Fig. 1a. They can be approximated as two-level systems that upon laser excitation at 532 nm show a broad emission at around 700 nm (see Fig. 1b). Other defects exist with different emission spectra [43, 44], and some of them have been successfully incorporated into nanodiamonds [45–49]. For instance, silicon-vacancy centers show a narrow emission spectrum centered at 740 nm and a linewidth of a few nanometers [32, 50, 51]. Defects can be produced by direct ion implantation with energies varying between few keV and MeV and posterior annealing at temperatures between 400 and 1200 °C [45, 52, 53]. In the case of color centers based on naturally abundant atoms in diamond, electron irradiation and annealing is used to mobilize vacancies [54, 55] in order to produce the right atomic configuration that leads to the fluorescent structures. In addition, nanodiamonds can also be grown by chemical vapor deposition (CVD) in a mixture of other gases generating the defect atom [56]. Once a defect is created in the diamond matrix, its fluorescent properties are extremely stable.

In this work, we used 35 nm diameter NDs acquired from Academia Sinica (brFND-35). Each ND contains on average 15 nitrogen-vacancy color centers. For these particular nanodiamonds nitrogen-vacancy (NV) centers exist in two different charges states: neutrally charged (NV0) and negatively charged (NV−) centers with zero-phonon lines at 575 and 637 nm, respectively, under 532 nm laser excitation (see Fig. 1c). We noted that the emission lies in the biological tissue window [57] and that the fluorescence of such defects present no blinking or photobleaching provided they are formed deeper than 2 nm from the surface [58–60].

### Functionalization of nanodiamonds

We functionalized the NDs’ surface with the R7-CLPFFD peptide (from here fNDs), a bi-functional peptide that confers different characteristics and functionalities to the NDs.

**Fig. 2:**
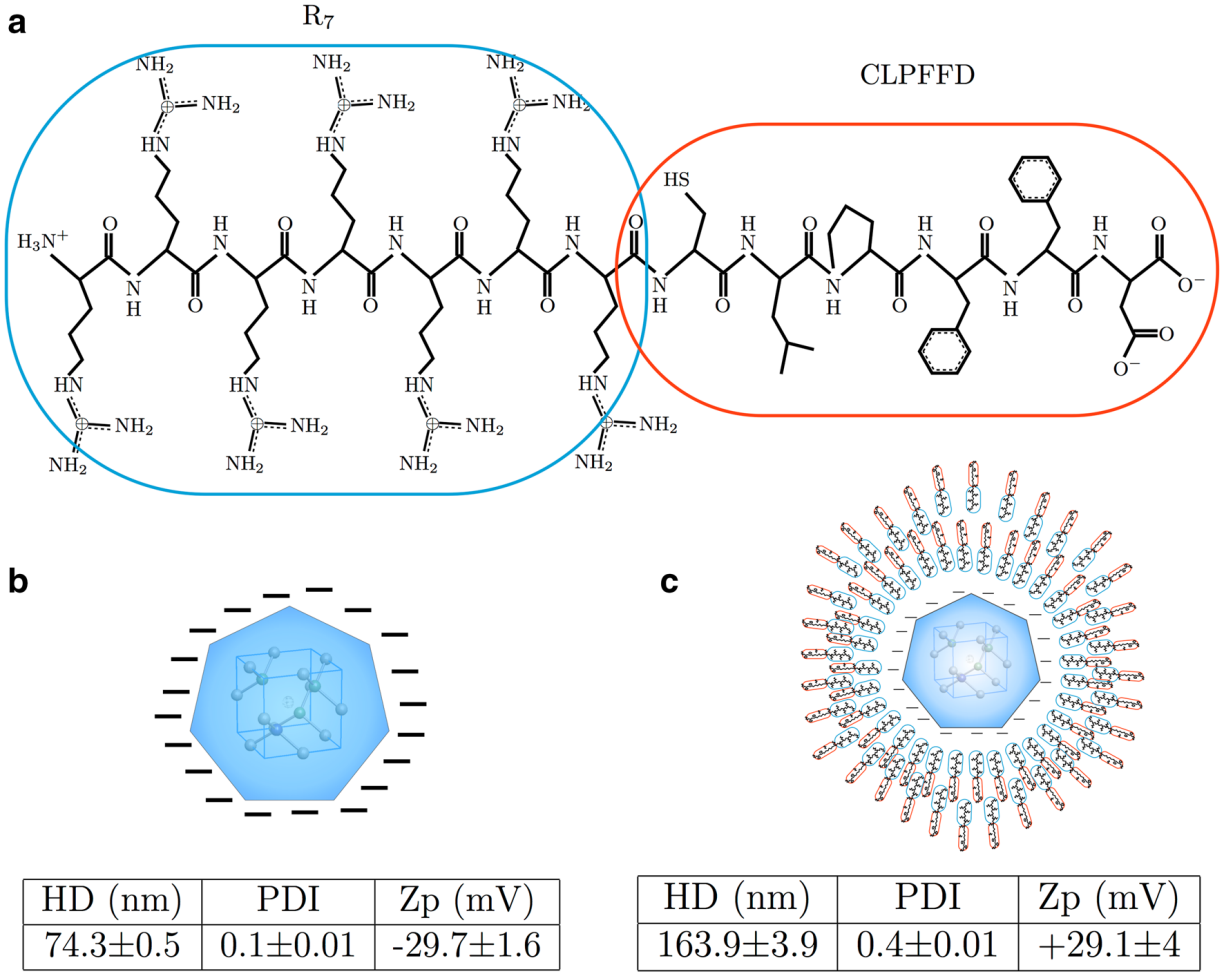
Functionalized nanodiamonds. **a** Bi-functional peptide composed of a cell penetrating R7 peptide (blue dotted area) that enhances the cellular uptake of its cargo, and a β-sheet breaker CLPFFD peptide (red dotted area) that recognizes toxic Aβ aggregates present in AD. **b** Zeta potential (Zp), hydrodynamic diameter (HD) and Polydispersity index (PDI) of naked and functionalized NDs. **c** Illustration and properties of fNDs

The R7-CLPFFD peptide is composed of two segments. The CLPFFD segment contains the native sequence of Aβ and has the ability to recognize Aβ aggregates [33, 61]. It includes hydrophobic residues Leu (L), Phe (F), and Phe (F), [31, 61–63] while the Asp residue (D) confers amphipathicity and a net charge of − 1 to the molecule (see Fig. 2a), increasing its solubility. This peptide is a modification of a peptide designed by Soto et al. [61] and has been used to stabilize, functionalize and enhance the brain targeting of gold nanoparticles [32, 62].

On the other hand the R7 segment, highly positive due to the presence of the arginine (secondary amines), was designed to promote cell penetration as it has been shown that the R7 peptide allows the crossing of cell membranes [34]. The R7 segment has also a net charge of + 7 allowing the electrostatic binding between the peptide and the negative charged surface of NDs (see Fig. 2) that contain carboxylate groups [64, 65]. Previously, both peptides have been independently used for functionalization of nanoparticles, transferring their properties to the nanocargo [61, 62, 66]. In addition, it was recently reported that, the affinity constant (Kd) of the LPFFD peptide for Aβ is 156 μM [67]. Finally, it has been demonstrated that this peptide, attached to gold nanospheres and gold nanorods, selectively binds to Aβ aggregates [32, 33, 68, 69].

To evaluate the adsorption of the R7-CLPFFD peptide to the surface of NDs we measured the Zeta potential (pZ) and a hydrodynamic diameter (Dh) of the fNDs in aqueous solution as an indicator of the electrostatic coating for this bi-functional peptide (see “NDs functionalization” section for further details on the functionalization procedure). First, the pZ value after peptide adsorption increased from 29.7 ± 1.6 to +29.1 ± 4.0 mV indicating that the nanocrystal was positively functionalized. Moreover, the increase in Dh from 74.3 ± 0.5 nm to 163.3 ± 2.0 nm, about twice the diameter of the bare nanocrystal, confirmed the functionalization. The pZ and Dh parameters of the fNDs remained stable after three washouts (see Fig. 2 and Additional file 1: Figures S1, S2, S3, S4 and S5 for further details).

Moreover the high-resolution transmission electron microscopy (HR-TEM) images of the fDNs compared to bare nanocrystals also indicate the capping with the peptide. The electronic density gradient is denser at the center than in the periphery of the particles indicating that the NDs are surrounded by peptides. The average particle diameter is 199 ± 56 nm, larger than the bare ND diameter. Moreover, it was possible to observe using HR-TEM that NDs are surrounded by a thick peptide layer, possibly forming a multilayer of peptides (see Additional file 1: Figure S6). On the other hand, the fluorescence spectrum of NDs did not change after functionalization and three consecutive washouts.

Altogether, these results support the successful adsorption of the R7-CLPFFD peptide onto the NDs surface. Previously, the surface of NDs has been successfully modified enabling applications such as luminescence imaging and drug delivery [43, 70–74] coupled with low toxicity and high fluorescent lifetime [75, 76]. NDs have been covalently and non-covalently coated with proteins such as streptavidin and glycoproteins, respectively [65]. As was previously reported, oxidation and reduction reactions have been used to terminate the surface of NDs with hydroxyl groups [77]. In addition, NDs have also been coated with peptides by silanization [78] and via electrostatic interactions [79].

Next, we evaluated the interaction of these R7-CLPFFD functionalized NDs (fND) with cells and assessed their properties as color markers.

### Cell internalization and ultrasensitive detection of functionalized ND

**Fig. 3:**
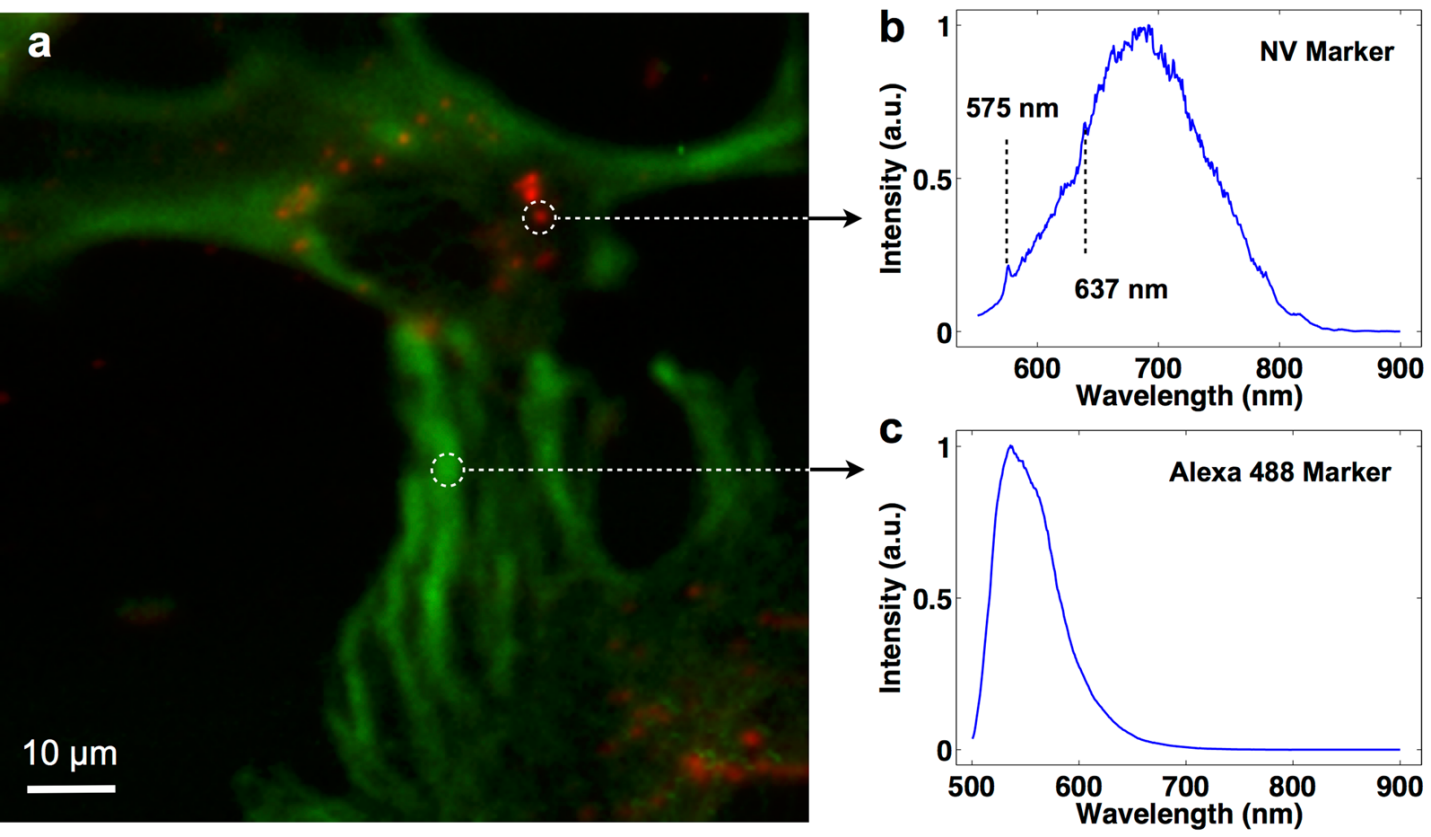
Cell internalization of functionalized NDs in a fibroblast cell line. **a** Merged image of fibroblast cells with Alexa 488 labeled tubulin excited with 488 nm laser illumination (green) and fNDs excited with 532 nm illumination (red). In both cases, the emission was recorded using an avalanche photon detector (APD). **b** Fluorescence spectrum of nanodiamonds showing the characteristic zero phonon lines at 637 and 575 nm. **c** Fluorescence spectrum of Alexa 488

The positive R7 region of the R7-CLPFFD peptide allows fNDs to cross the cell membrane because of its cell penetrating (CPP) function [80, 81]. To evaluate fNDs internalization, fibroblasts (30.000 cells/ml) were incubated with 8 pM fNDs. After 6 h the samples were washed and fixed. In order to visualize the cells, microtubules (cytoskeleton components) were immunostained using anti-β-tubulin antibodies (1:1000) and secondary antibodies conjugated with Alexa Fluor 488 (1:1000). The samples were analyzed in a home-built confocal microscope setup equipped with an avalanche photon detector (APD) and an optical spectrometer (see Additional file 1 for further information). Figure 3a shows a representative fibroblast cell image incubated with fNDs. First, due to the high sensitivity of the APD, this setup allowed us to use NDs concentrations in the pM range and we were even able to detect NDs containing single emitters. Second, the analysis of different regions of interest (ROIs) of the sample clearly shows two distinguishable spectra: one corresponding to the Alexa Fluor 488, and the other corresponding to the fND (see Fig. 3b, c, respectively). The fNDs and Alexa 488 were observed in the same focal plane.

Interestingly, Fig. 3a shows fNDs inside of the perimeter of the cell whereas no fNDs were observed outside the cell (washed samples) suggesting that fNDs are inside the cell. In addition, no fNDs were observed in the cell nucleus.

**Fig. 4:**
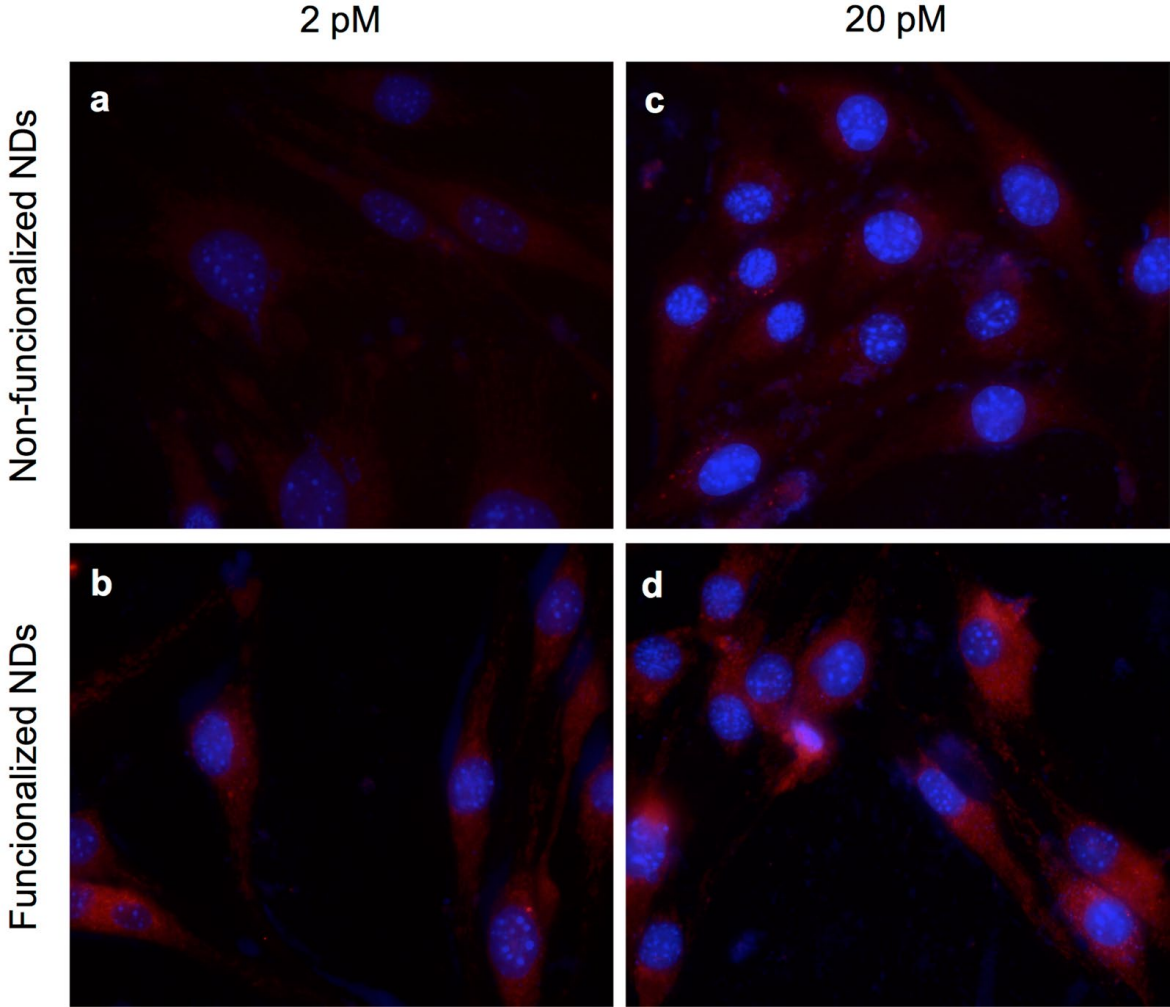
Cell internalization of functionalized NDs in the bEnd.3 cell line. Image of bEnd.3 cells incubated with ND (panels **a** and **c**) and fND (panels **b** and **d**) at concentrations of 2 pM (panels **a** and **b**) and 20 pM (panels **c** and **d**) for 6 h

After evaluating cell internalization of fNDs by analyzing their emission spectrum, we further evaluated the cell penetration capacity of fNDs in a cell model more closely related to the biological context of the blood–brain barrier. The bEnd.3 cells are brain vascular endothelial cells and are commonly used in different in vitro models of BBB drug transportation. bEnd.3 cells were incubated at nanodiamond concentrations of 2 and 20 pM using both functionalized and non-functionalized nanodiamonds as a control. Figure 4 shows representative images of cells after a 6 h incubation. The penetration of functionalized nanoparticles (red signal) into bEnd.3 cells is increased (bottom panel) in comparison to non-functionalized nanoparticles (top panel).

**Fig. 5:**
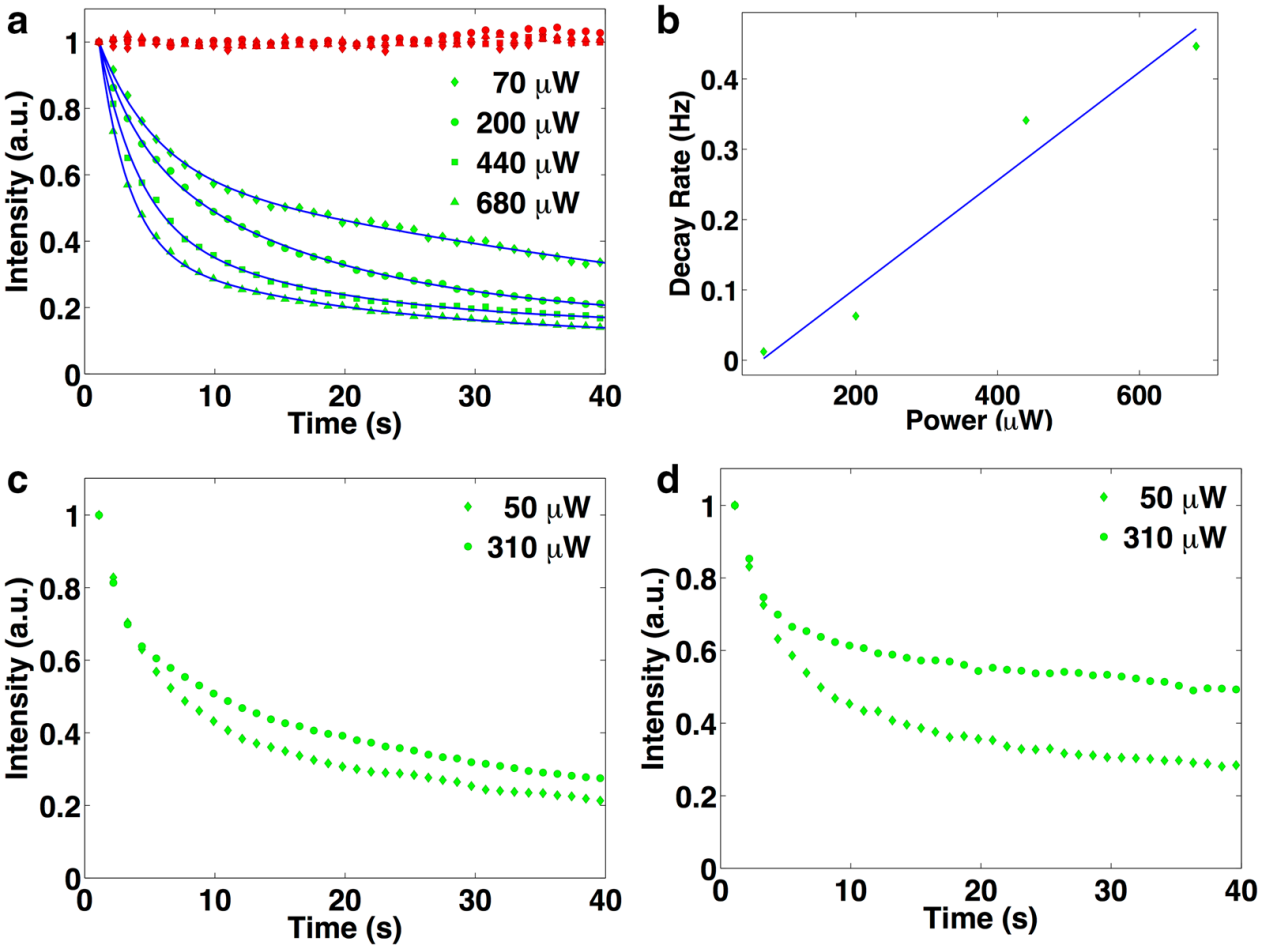
Characterization of the photo stability of diamond-based color marker and Alexa Fluor 555-conjugated. **a** Fluorescence traces under continuous 532 nm wavelength laser illumination of Alexa Fluor 555-conjugated (green marker) and fNDs containing nitrogen-vacancy color centers (red marker) for several laser powers. **b** Decay rate of Alexa Fluor 555-conjugated versus the excitation power. The fluorescence decreases its intensity at a rate of 0.8 Hz/mW whereas that of the fNDs remained steady. **c** Fluorescence intensity vs. time illumination of Alexa Fluor 488 and (**d**) FITC

In order to evaluate the fluorescence stability of fNDs, we compared its fluorescence intensity to that of Alexa Fluor 555 through the monitoring of the samples over 5 min under several excitation wavelengths. At different laser powers we observed that Alexa Fluor 555 fluorescence decreased over time at a rate of 0.8 Hz/mW (see Fig. 5a, b). Figure 5c, d also shows the fluorescence stability of Alexa 488 and FITC respectively, under several laser excitation powers. In contrast, the fluorescence of fNDs remained constant. Therefore, diamond based fluorescent markers are more stable than other fluorescent markers. The fluorescence shows no noticeable decay after several days under laser excitation, and after months, or even years without continuous laser excitation, enabling long-term experimentation.

**Fig. 6:**
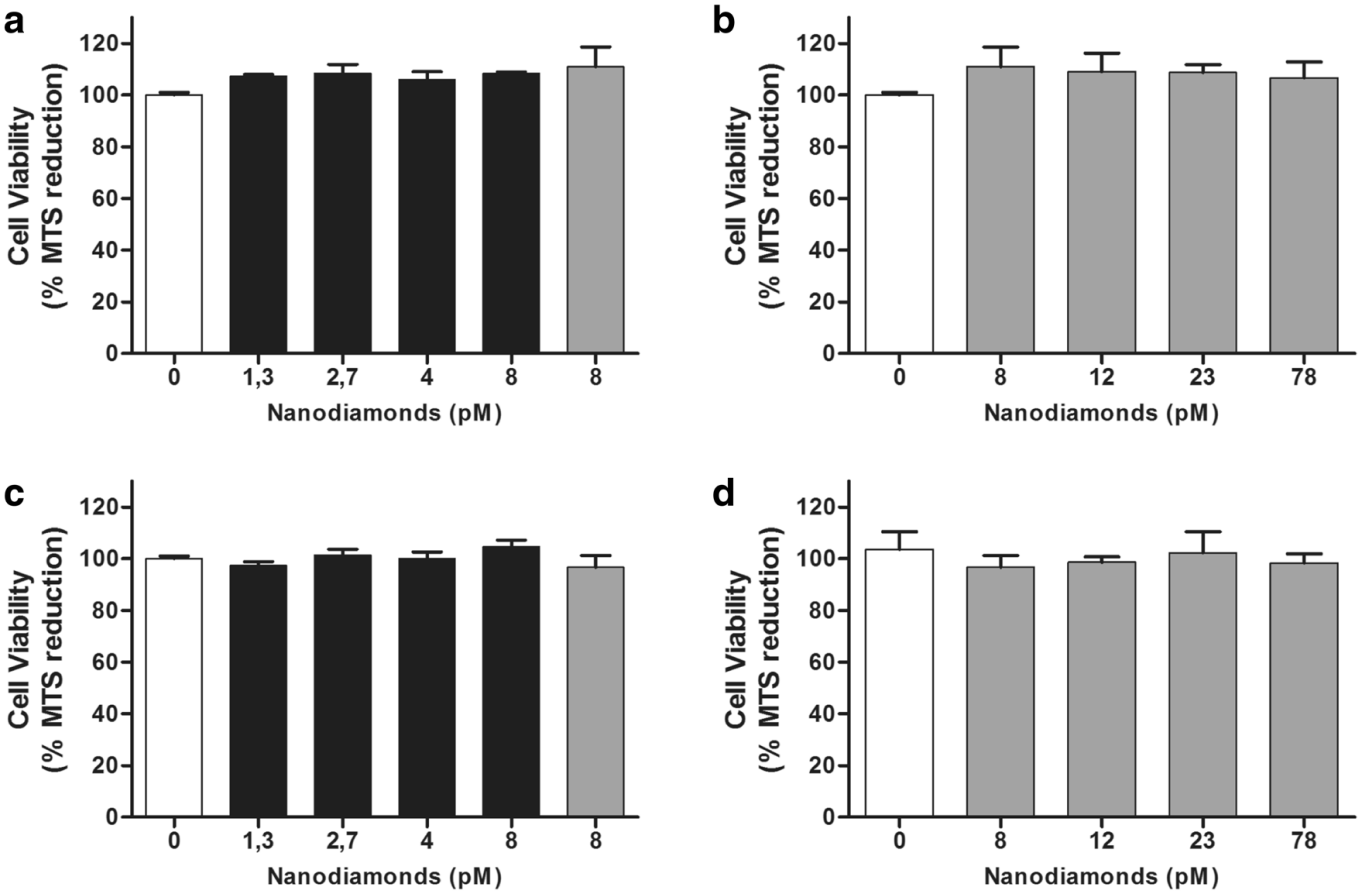
The functionalization of NDs does not affect cell viability. Cell viability measurements evaluated with the MTS reduction assay in **a** HT22 and **c** C3 10T1/2 cell lines incubated with different concentrations of fNDs (black bars) for 24 h and non-functionalized NDs (grey bars). Further tests under higher concentrations of non-functionalized nanodiamonds were performed for **b** HT22 and **d** C3 10T1/2 cell lines. Values correspond to the mean percentage of viable cells with respect to the control cells (white bars). Error bars indicate standard deviation estimated from three experiments each carried out in triplicate

Next we tested the effects of fNDs on cell viability. Although NDs have been described as biocompatible nanoparticles [82], there are a few studies showing that some types of NDs may have a negative impact on cell viability [83, 84]. Moreover, one study even suggests that NDs might have bactericidal properties depending on their surface termination [85]. Therefore, we tested fNDs viability on two different cell lines using the MTS assay (see Additional file 1). The MTS test is based on estimating MTS tetrazolium reduction by cellular respiration of viable cells, which generates a purple colored formazan product that can be quantified at 490 nm. The percentage of MTS reduction is an indicator of cell viability. We incubated HT22 hippocampal neurons and 3T3 fibroblast cells with different concentrations of fNDs for 24 h. The treated cells showed no significant differences in cell viability compared to the control groups (see Fig. 6). In addition, we did no find significant differences in cell viability using higher concentration of non-functionalized NDs in either cell line. Therefore, the physical and chemical properties of ND and fNDs do not affect cell viability.

This would be one of the main advantages of our cellular marker nanosystem compared with quantum dots, which are highly toxic under certain conditions [86, 87].

### Binding of fNDs to Aβ fibers

**Fig. 7:**
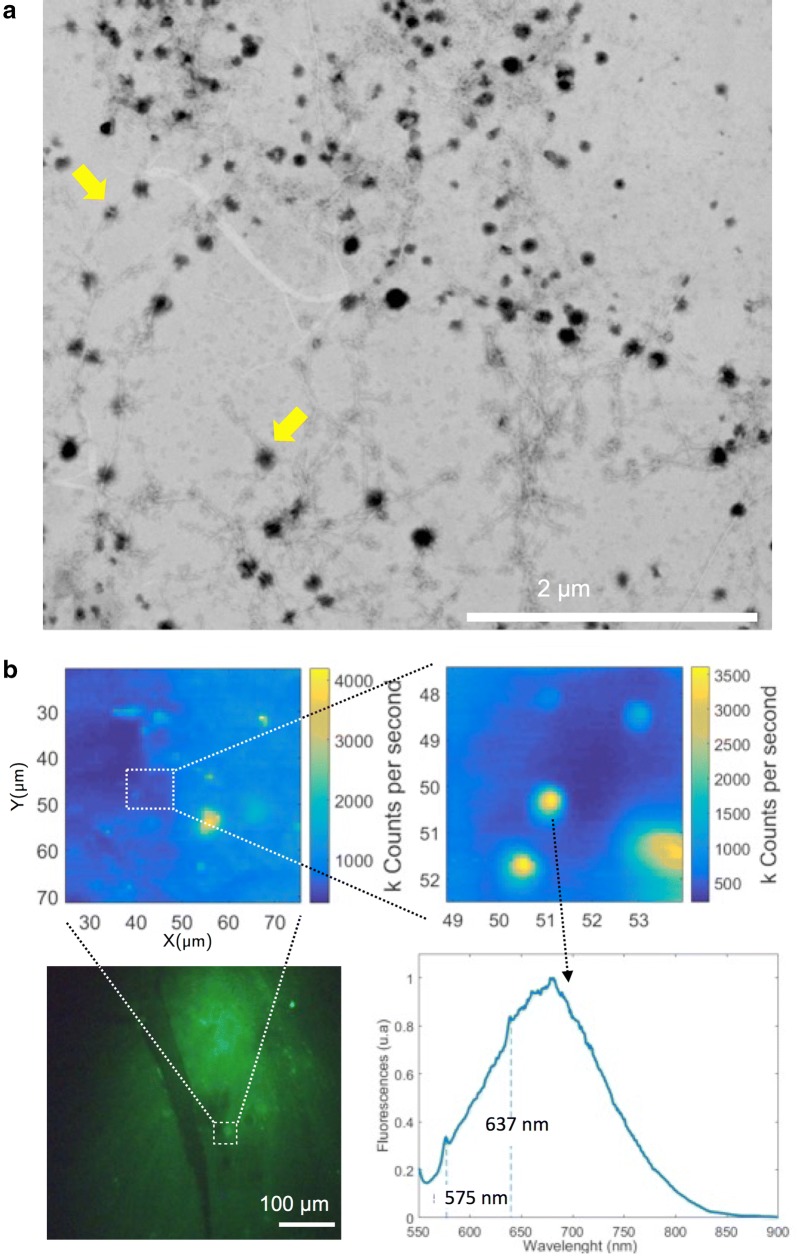
Association of fNDs with Aβ fibers and plaques. **a** STEM image showing fibers of Aβ and fNDs together (yellow arrows show two specific fNDs, as examples). Regions without fibers show almost no fNDs. **b** The image shows the hippocampus of AD mouse brain tissue slides stained for detection of Aβ plaques with the anti Aβ antibody 4G8 and Alexa 488 (green) secondary antibody; and zoomed images showing fNDs illuminated with 532 nm laser. The first inset shows a confocal image zoom of 50 × 50 um2 near an Aβ plaque. The second inset shows a confocal image zoom 5 × 5 um2. Finally, a typical emission spectrum of a fND detected under 532 nm excitation near an Aβ plaque is shown

Then, we performed in vitro experiments to evaluate the ability of fNDs to bind Aβ fibrilar aggregates. Aβ fibers were grown in vitro and then incubated with fNDs under constant stirring for 30 min. The binding of fNDs to Aβ fibers was evaluated by scanning transmission electron microscopy (STEM). Figure 7a shows that fNDs co-localize with Aβ fibers, decorating the fibrilar aggregates. Almost no fNDs are observed in regions without fibers. We performed a control assays incubating fibrilar albumin aggregates with fNDs. We did not observed any interaction between these aggregates and fNDs (see Additional file 1: Figure S7). These observations suggest a specific interaction between fNDs and Aβ fibers, probably due to the CLPFFD region of the bifunctional R7-CLPFFD peptide on the fNDs surface.

Since it is possible that the interaction between fNDs and Aβ trough the CLPFFD region could affect the Aβ aggregation process, we performed an Aβ in vitro aggregation assay in the presence of fNDs. We found that fNDs inhibited fibril formation by 40% (see Additional file 1: Figure S8). Altogether these results indicate that fNDs could be able to attach to Aβ aggregates.

On the other hand, in order to visualize the association between fNDs and Aβ plaques we incubated slices of brain tissues from AD transgenic mice that overexpressed Aβ with fNDs. The slices were also co-incubated with the 4G8 antibody (antibody against Aβ) and then with a secondary antibody conjugated with Alexa 488 for visualization of Aβ plaques. Figure 7b shows the association between both fluorescent signals (fNDs and the 488 signals) in the halo of Aβ plaques, indicating detection of Aβ peptide by the two markers. In regions where no Aβ plaques are present, we did not find fNDs (see Additional file 1: Figure S9). Currently, one of the most used staining methods for Aβ aggregates is Thioflavin S (ThS) [33, 88], a fluorescent marker with a short fluorescence lifetime when bound to amyloid fibers [89]. Therefore, fNDs can be used as fluorescent probes to detect regions of Aβ aggregates.

These results suggest that fNDs can become a powerful method for studying the formation of Aβ aggregates during AD development. We showed that fNDs could be internalized in two cellular models, fibroblast cells and in the cell line, resulting in no fNDs in the region between cells. bEnd.3 is a brain vascular endothelial cell line commonly used as an in vitro model for transportation through the blood–brain barrier. This result is relevant from the point of view of AD treatment and diagnosis. The bi-functional conjugated peptide can serve as a biodetector of extracellular Aβ fibrils (once these nanosystems attach to Aβ aggregates thanks to its CLPFFD peptide region) and also has the ability to cross biological barriers favored by its oligoarginine region, which may promote its delivery to the brain parenchyma. In this way, the evaluation of the characteristic emission spectra of NDs fluorescence in the extracellular region, where the amyloid aggregates form in the CNS, may be used as an indicator of the presence of Aβ fibrils. The remarkable fluorescence stability of the NDs emission would allow for reliable biodetection of Aβ in longterm experiments. Therefore, fNDs offer the opportunity to track the progression of AD.

## Conclusions

We have shown that the surface of nanodiamonds containing stable fluorescent color centers can be functionalized to perform non-trivial and multiple tasks without damaging the stability of their fluorescence. This remarkable stability allows for the detection of NDs at as low as picomolar concentrations using confocal microscopy. In particular, this peptide functionalization may be used in reliable and long-term experiments for detecting Aβ aggregates and follow their formation. Finally, we showed that fNDs penetrate endothelial cells used as models to test the crossing of the BBB and do not affect cell viability in the two cell lines considered in this work. Therefore, NDs have important advantages over common fluorescent markers or quantum dots.

## Experimental section

### Confocal setup

We used a home built confocal microscope with an air objective of 0.9 numerical aperture (NA) and 1 mm working distance (WD) (Nikon TU Fluor Plan 100×). For the simultaneous observation of a larger field image of the brain and detection of NVC, we added an air 40 × objective connected to de camera on the face of the 100× objective. The excitation was provided by a 532 nm green laser and with 0.5 mW power. The fluorescence was filtered by a dichroic mirror at 532 nm (BrightLine laser dichroic beam splitter) and recorded using an avalanche photon detector (Perkin Elmer SPCM-140-ARQM) and a 532 nm notch filter. In addition, part of the fluorescence was directed to a spectrometer (QE Pro Spectrometer, OceanOptics). Images were generated by scanning the sample with a high precision XYZ piezo (Modular Piezo-Controller E501, Physik Instrumente).

### Synthesis of the peptide

The R7-CLPFFD peptide was synthesized using a fluorenylmethyloxycarbonyl (Fmoc) strategy in solid-phase synthesis as a C-terminal amide. The side-chain of cysteine was protected with the trityl group, which is removed during the final cleavage to render the free thiol. Fmoc-protected amino acids were purchased from Iris Biotech (Marktredwitz, Germany). In addition, 1-[bis(dimethylamino) methylene]-1H-benzotriazolium tetrafluoroborate 3-oxide (TBTU), FmocAM handle, and resin MBHA were obtained from Novabiochem. The chemical reagents N,N′-diisopropylcarbodiimide (DIPCI), 1-hydroxybenzotriazole (HOBt), triethylsilane (TES), and N,N’-dimethylaminopyridine (DMAP) were obtained from Fluka (Buchs, Switzerland). Manual synthesis included the following steps: (i) resin washing with DMF (5 × 30 s), (ii) Fmoc removal with 20% piperidine/DMF (1 × 1 min + 2 × 7 min), (iii) washing with DMF (5 × 30 s), (iv) washing with DMF (5 × 30 s) and CH2 Cl2 (5 × 30 s), (v) Kaiser’s test (with a peptide-resin sample), and (vi) washing with DMF (5 × 30 s). Peptides were cleaved by acidolysis with trifluoroacetic acid (TFA) using TIS, 2,2′-(ethylenedioxy)- diethanethiol (DOTA) water as scavengers (92.5:2.5:2.5)(v/v/v) for 90 min. TFA was removed with a N2 stream, and the oily residue was precipitated with dry tert-butyl ether. Crude peptides were recovered by centrifugation and decantation of the ethyl ether phase. The pellet was redissolved in 10% acetic acid (HOAc) and lyophilized. The peptide was analyzed using RP-HPLC with a Waters 996 photodiode array detector (λ 443 nm) equipped with a Waters 2695 separation module (Milford, MA), a Symmetry column (C18, 5 µm, 4.6 × 150 mm), and Millennium software at a flow rate (1 ml/min, gradient) of 5–100% B over 15 min (A) 0.045% TFA in H2 O, and (B) 0.036% TFA in acetonitrile. The peptide was purified using semipreparative RP-HPLC with a Waters 2487 Dual Absorbance Detector equipped with a Waters 2700 Sample Manager, a Waters 600 Controller, a Waters Fraction Collector, a Symmetry column (C18, 5 µm, 30 × 100 mm), and Millennium software. The peptide was finally analized with MALDI-TOF with a Bruker model Biflex III. Using MALDI-TOF, the R7-CLPFFD (H-R7CLPFFD-NH2) [M+Na+]: 1834.05 peptide was identified (see Additional file 1: Figure S10).

### NDs functionalization

The RRRRRRRCLPFFD peptide was dissolved in ultra pure Milli Q water at a final concentration of 0.05 mg/ml. The nanodiamonds are added to this peptide solution remaining at a concentration of 0.8 nM in the final solution, and then they are incubated with vigorous stirring for 2 h. The adsorption of the peptide on the nanocrystal surface was evaluated by the change in the Zeta potential (pZ) and hydrodynamic diameter (Dh) (Zeta sizer 3000, Malvern Instruments, UK). The colloidal suspension was centrifuged and washed three times. The washed fNDs were reevaluated by Zp and Dh to ensure that the functionalization remained. Finally, we analyzed the functionalization of the nanodiamond by High Resolution Transmission Electron Microscopy (HR-TEM) staining the samples with phosphotungstic acid (1%) in order to evaluate the presence of the peptide surrounding the nanodiamond.

### Amyloid fibers

Aβ1-42 was purchased from r-Peptide. Aβ was dissolved in water, aliquoted, lyophilized and stored in glass vials at − 20 °C until used. To obtain mature Aβ fibrils, the aliquots were treated with 1,1,1,3,3,3-hexafluoro-2-propanol (HFIP) for 30 min to obtain the monomeric Aβ form. Aliquots were then lyophilized and resuspended in GNR-CLPFFD solution (0.2 nM approx.). The final Aβ concentration was 20 μM. The samples were incubated for 3 days at 37 °C with mechanical shaking. For STEM observations, the samples were adsorbed for 1 min onto glow discharged holey carbon films on 200 mesh copper grids. The TEM grids were then blotted and washed in Milli-Q water before negative staining with 1% phosphotungstic acid for visualization by STEM.

### Culture cells

HT22 cells were kindly donated by Elena Pasquale (Sanford-Burnham Medical Research Institute, La Jolla, California, United States of America) and 3T 1/2 cells were kindly donated by Enrique Brandan (P. Universidad Católica de Chile, Santiago, Chile). HT22 and C3H 10T1/2 cells were maintained in Dulbecco’s modified Eagle’s medium (DMEM) supplemented with 10% fetal bovine serum, 100 IU/ml penicillin, and 100 μg/ml streptomycin.

### Immunofluorescence assay

Cells were rinsed twice with PBS, fixed with 4% paraformaldehyde in PBS for 20 min, and permeabilized for 10 min with 0.2% Triton X-100 in PBS. After rinsing twice with PBS, the cells were incubated in 3% BSA in PBS for 30 min at room temperature, followed by an overnight incubation at 4 °C with primary antibodies against β-Tubulin (Santa Cruz Biotechnology). The cells were washed four times with PBS and then incubated with anti-rabbit Alexa 488 antibodies (Life Technologies) for 1 h at room temperature.

### Cell penetration in the bEnd.3 cell line

Brain vascular endothelial cells (murine bEnd.3 cells, ATCC CRL-2299) were grown following the supplier instructions in DMEM with 4.5 g/L glucose, 3.7 g/L sodium bicarbonate, 4 mM glutamine, 10% FBS, 100 U/ml penicillin and 100 μg/ml streptomycin. Cells were maintained in a humidified cell culture incubator at 37 °C and 5% CO_2_. After reaching confluence, cells were trypsinized and seeded at a density of 1.0 × 10^5^ cells in polylysine coated coverslips. After 24 h, increasing nanoparticles concentrations (2 and 20 pM) were applied to the coverslips and incubated for 6 h. Then, cells were washed with PBS, fixed with paraformaldehyde and the nuclei stained with DAPI and visualized witn an Olympus BX51 fluorescence microscope.

### Cell viability assay

For this test we used the embryonic fibroblast cell line C3H 10T1/2 and HT22 cells. The cells were seeded in 96-well plates at 5 × 103 cell/100 ml per well and maintained en supplemented DMEM medium. Then, cells were incubated with functionalized Nanodiamonds for 24 h. Cell viability was measured using the [3-(4,5-dimethylthiazol-2-yl)-5-(3-carboxymethoxyphenyl)-2-(4-sulfophenyl)-2H-tetrazolium (MTS) assay (Mossman, 1983). After a 2 h incubation with MTS, a purple color developed within the cells, indicating the cleavage of the tetrazolium salt (MTS) by mitochondrial reductase in live cells. The purple product (formazan products that are directly soluble in cell culture medium) was measured at 492 nm using an enzyme-linked immunosorbent assay (ELISA) reader (Autobio PHomo). The percent reduction of MTT was compared to controls cells not exposed to the material, which represented 100% MTT reduction.

### Incubation of fND with amyloid fibrils

Aβ1-42 was purchased from r-Peptide. Aβ was dissolved in water, aliquoted, lyophilized and stored in glass vials at − 20 °C until used. To obtain mature Aβ fibrils, the aliquots were treated with 1,1,1,3,3,3-hexafluoro-2-propanol (HFIP) for 30 min to obtain the monomeric Aβ form. Aliquots were then lyophilized and resuspended in fND solution (0.5 nM approx.). The final Aβ concentration was 20 μM. The samples were incubated for 2 days at 37 °C with mechanical shaking. After that, we used the Thioflavin-T assay to detect amyloid fibrils, and the samples were placed in a black 96-well plate with 0.5 M glycine buffer, pH 8.4 and 0.1 M Thioflavin-T. The samples were measured with excitation at 450 nm and emission at 480 nm.

### Interaction of fND with amyloid fibrils

The amyloid fibrils were incubated for 30 min with a solution of fND (0.5 nM). After that, TEM grids were blotted and washed in Milli-Q water before negative staining with 1% phosphotungstic acid for visualization by STEM.

### Interaction of fND with albumin fibrils

Bovine serum albumin (BSA) was dissolved in 10 mM phosphate buffer pH 7.4 with 10 mM NaCl. The samples were incubated for 5 days at 65 °C with mechanical shaking at 300 rpm. The fibrils were mixed with a solution of fND (concentration: 30 µM for the protein and 0.5 nM for the fND) and incubated for 30 min. For STEM observations, the samples were adsorbed for 1 min onto glow discharged holey carbon films on 200 mesh copper grids. The TEM grids were then blotted and washed in Milli-Q water before negative staining with 1% phosphotungstic acid for visualization by STEM.

### Immunolabelling of floating sections

Transgenic 12-month-old APPswe/PSEN1dE9 mice (The Jackson Laboratory) were anesthetized and perfused with 30 ml of ice-cold PBS and then with 4% paraformaldehyde in PBS. Brains were removed and postfixed at 4 °C overnight, followed by 20 and 30% sucrose in PBS at 4 °C overnight. Brains were cut into 30 μm coronal sections with a cryostat (Leitz 1900) at − 20 °C. Immunolabelling was performed using the anti-Aβ 4G8 antibody (1:100, Biolegend, CA). Anti-mouse-IgG conjugated with Alexa Fluor-488 (1:1000, Molecular Probes), were used as secondary antibodies. Finally, sections were washed four times for 10 min with PBS and then incubated with fNDs for 1 h at a concentration 0.1 nM. The sections were then washed four times for 10 min with PBS and mounted with mounting medium DAKO.

## Additional file

**Additional file 1: Figure S1.** Size distribution by intensity measured by DLS. (A) bare nanodiamonds and (B) funtionalized nanodiamonds. **Figure S2.** Size distribution by number measured by DLS. (A) bare nanodiamonds and (B) funtionalized nanodiamonds. **Figure S3.** Size distribution by volume measured by DLS. (A) bare nanodiamonds and (B) funtionalized nanodiamonds. **Figure S4.** Z-potential values. (A) bare nanodiamonds and (B) funtionalized nanodiamonds. **Figure S5.** AFM characterization oft he size of nanodiamonds. Left: AFM image of non-functionalized nanodiamonds. Right: Histogram oft he size of nanodiamonds estimated from the AFM image. **Figure S6.** Structure of nanodiamonds. HR-TEM. Electron micrographs showing (A) NDs and (B) fNDs. **Figure S7.** Association of fNDs with albumin fibers. STEM Images. fNDs were incubated with albumin fibers to evaluate an unspecific interaction of fNDs with another kinds of fibers. No interaction between albumin fibers and fNDs was observed. Free fNDs (A) and albumin fibers (B) were observed separately. **Figure S8.** Thioflavin-T results. Fluorescence intensity signal from samples of Aβ-amyloid fibrils in presence of 0.5 nM of fND. The results are expressed as percentages with respect to the intensity from Aβ-amyloid fibrils free peptide. **Figure S9.** Association of fNDs with Aβ fibers and plaques. Composed confocal image of AD mouse brain tissue slides stained to detect Aβ plaques with an anti Aβ antibody 4G8 and Alexa 488 (green points) secondary antibody; and fNDs illuminated with 532 nm laser. Inset: zoom outside the neighborhood of Aβ plaque where we are not able to detect fNDs. **Figure S10.** HPLC chromatogram and MS/MALDI TOF spectrum of the R7CLPFFD. The HPLC chromatogram was realized with a column Kromasil 100-5C18 (250 × 4.6 mm), using a gradient 0–40% acetonitrile whit a retention time of 30 min and λ of detection was 200 nm. Mass spectra were performed using a matrix of 2,5-dihidrobenzóico acid (DHB) and α-cyano-4-hydroxycinnamic acid (ACH) at a concentration of 10 mg/ml in acetonitrile/formic acid 0.1% v/v (1:2)

## Abbreviations

NDs: stable fluorescent markers based on nanodiamonds
Aβ: amyloid β
GFP: Green Fluorescent Protein
FRET: fluorescence resonance energy transfer
QD: quantum dots
NPs: nanoparticles
EPR: Enhanced Permeability and Retention
AD: Alzheimer’s disease
fNDs: functionalized NDs
R7: RRRRRRR
CPP: cell penetrating peptide
BBB: brain–blood barrier
NV: nitrogen-vacancy
CVD: chemical vapor deposition
NV0: neutrally charged NVs
NV−: negatively charged NV
pZ: Zeta potential
Dh: hydrodynamic diameter
HR-TEM: high-resolution transmission electron microscopy
STEM: scanning transmission electron microscopy
APD: avalanche photon detector
ThS: Thioflavin S
