# Supplementary material for: Functionalization of stable fluorescent nanodiamonds towards reliable detection of biomarkers for Alzheimer’s disease
Source: J Nanobiotechnology. 2018 Aug 10;16:60. doi: 10.1186/s12951-018-0385-7 (PMC6085760; doi:10.1186/s12951-018-0385-7)
Supplement: Supplementary file 1 — Additional file 1: Figure S1. Size distribution by intensity measured by DLS. (A) bare nanodiamonds and (B) funtionalized nanodiamonds. Figure S2. Size distribution by number measured by DLS. (A) bare nanodiamonds and (B) funtionalized nanodiamonds. Figure S3. Size distribution by volume measured by DLS. (A) bare nanodiamonds and (B) funtionalized nanodiamonds. Figure S4. Z-potential values. (A) bare nanodiamonds and (B) funtionalized nanodiamonds. Figure S5. AFM characterization oft he size of nanodiamonds. Left: AFM image of non-functionalized nanodiamonds. Right: Histogram oft he size of nanodiamonds estimated from the AFM image. Figure S6. Structure of nanodiamonds. HR-TEM. Electron micrographs showing (A) NDs and (B) fNDs. Figure S7. Association of fNDs with albumin fibers. STEM Images. fNDs were incubated with albumin fibers to evaluate an unspecific interaction of fNDs with another kinds of fibers. No interaction between albumin fibers and fNDs was observed. Free fNDs (A) and albumin fibers (B) were observed separately. Figure S8. Thioflavin-T results. Fluorescence intensity signal from samples of Aβ-amyloid fibrils in presence of 0.5 nM of fND. The results are expressed as percentages with respect to the intensity from Aβ-amyloid fibrils free peptide. Figure S9. Association of fNDs with Aβ fibers and plaques. Composed confocal image of AD mouse brain tissue slides stained to detect Aβ plaques with an anti Aβ antibody 4G8 and Alexa 488 (green points) secondary antibody; and fNDs illuminated with 532 nm laser. Inset: zoom outside the neighborhood of Aβ plaque where we are not able to detect fNDs. Figure S10. HPLC chromatogram and MS/MALDI TOF spectrum of the R7CLPFFD. The HPLC chromatogram was realized with a column Kromasil 100-5C18 (250 × 4.6 mm), using a gradient 0–40% acetonitrile whit a retention time of 30 min and λ of detection was 200 nm. Mass spectra were performed using a matrix of 2,5-dihidrobenzóico acid (DHB) and α-cyano-4-hydroxycinnamic acid (AC [file 12951_2018_385_MOESM1_ESM.docx]

Additional Material

Functionalization of stable fluorescent nanodiamonds towards reliable detection of Alzheimer biomarkers

Francisco Morales-Zavala^1,2§^, Nathalie Casanova-Morales^3§^, Raúl B. Gonzalez^3^, América Chandía-Cristi^4^, LD Estrada^5^, Ignacio Alvizú^3^, Victor Waselowski^3^, Fanny Guzman^6^, Simón Guerrero^1,2^, Marisol Oyarzún-Olave^4^, Cristian Rebolledo^3^, Enrique Rodriguez^3^, Julien Armijo^3^, Heman Bhuyan^3^, Mario Favre^3^, Alejandra R. Alvarez^4,7,*^, Marcelo J. Kogan^1,2,*^, Jerónimo R. Maze^3,7*^

^1^Department of Pharmacological and Toxicological Chemistry, Facultad de Ciencias Químicas y Farmacéuticas, Universidad de Chile,

^2^Advanced Center for Chronic Diseases (ACCDiS), Santiago, Chile

^3^Institute of Physics, Pontificia Universidad Católica de Chile, Santiago, 7820436, Chile

^4^Department of Cell & Molecular Biology, Pontificia Universidad Católica de Chile, Santiago, Chile

^5^Centro Integrativo de Bilogía y Química Aplicada, Universidad Bernardo O’Higgins, Santiago, Chile.

^6^Núcleo de Biotecnología Curauma, Pontificia Universidad Católica de Valparaíso, Chile

^7^Center for Nanoscale Technology and advanced Materials, Pontificia Universidad Catolica de Chile, Santiago, Chile.

§These authors contributed equally to this work

E-mail: [jmaze@uc.cl](mailto:jmaze@uc.cl), [mkogan@ciq.uchile.cl](mailto:mkogan@ciq.uchile.cl), [aalvarez@bio.puc.cl](mailto:aalvarez@bio.puc.cl)

Keywords: Fluorescent markers, nanodiamonds, peptide R7-CLPFFD, Alzheimer disease, Amyloid beta.

**Size characterization of nanodiamonds**

Using dynamic light scattering (DLS) we characterize the size distribution by intensity, by number and by volume, of non-functionalized nanodiamonds (NDs) and functionalized nanodiamonds (fNDs).


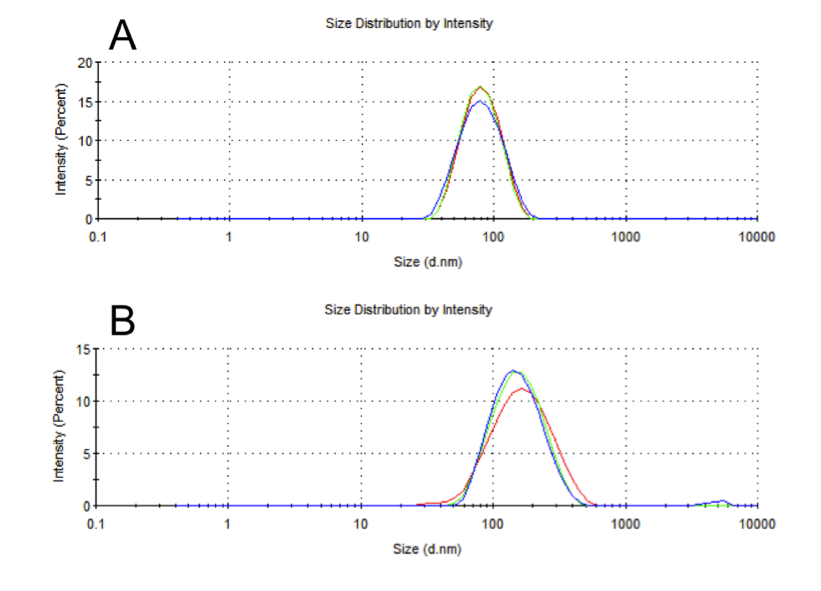


**Figure S1. Size distribution by intensity measured by DLS.** (A) bare nanodiamonds and (B) funtionalized nanodiamonds.


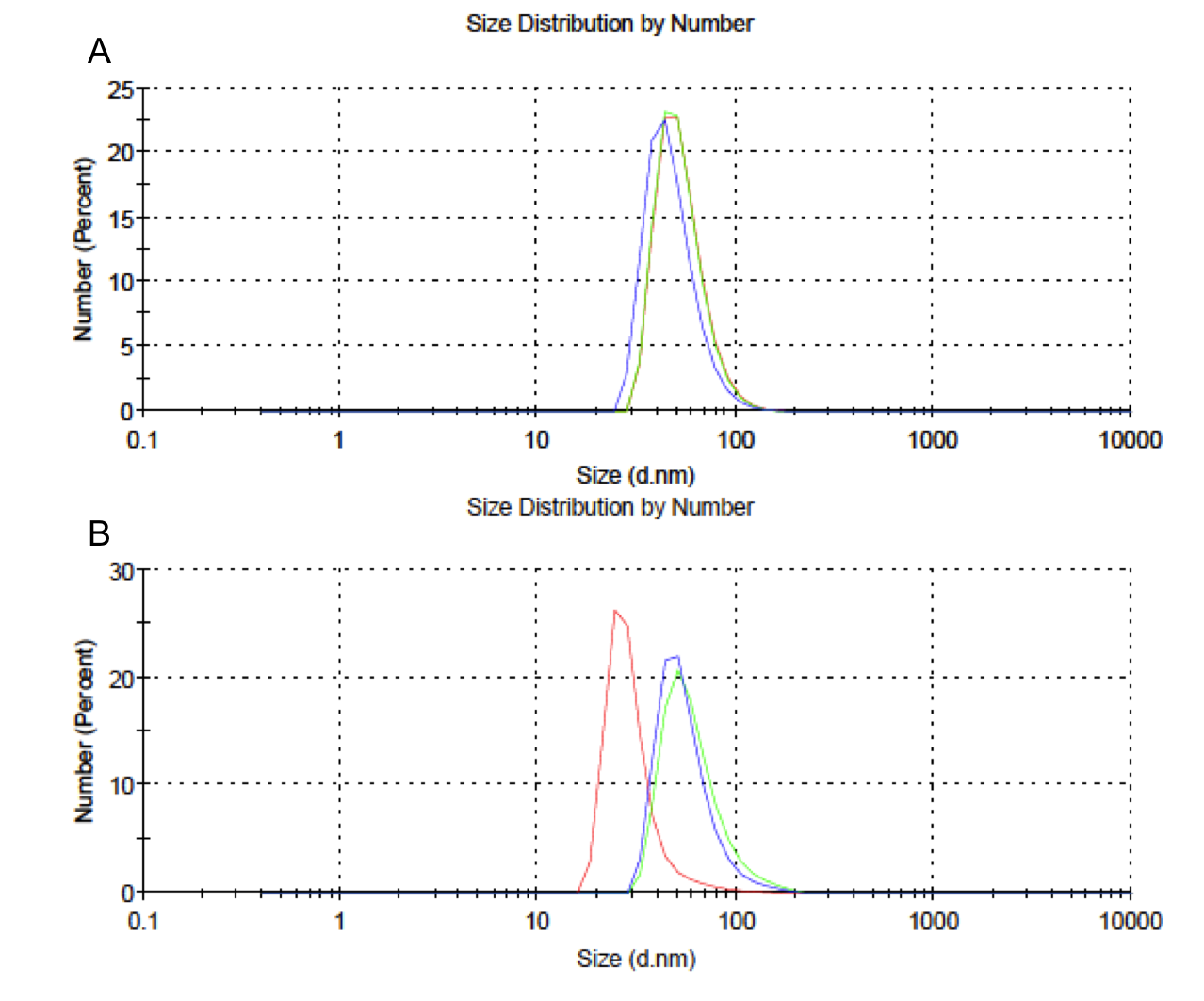


**Figure S2. Size distribution by number measured by DLS.** (A) bare nanodiamonds and (B) funtionalized nanodiamonds.


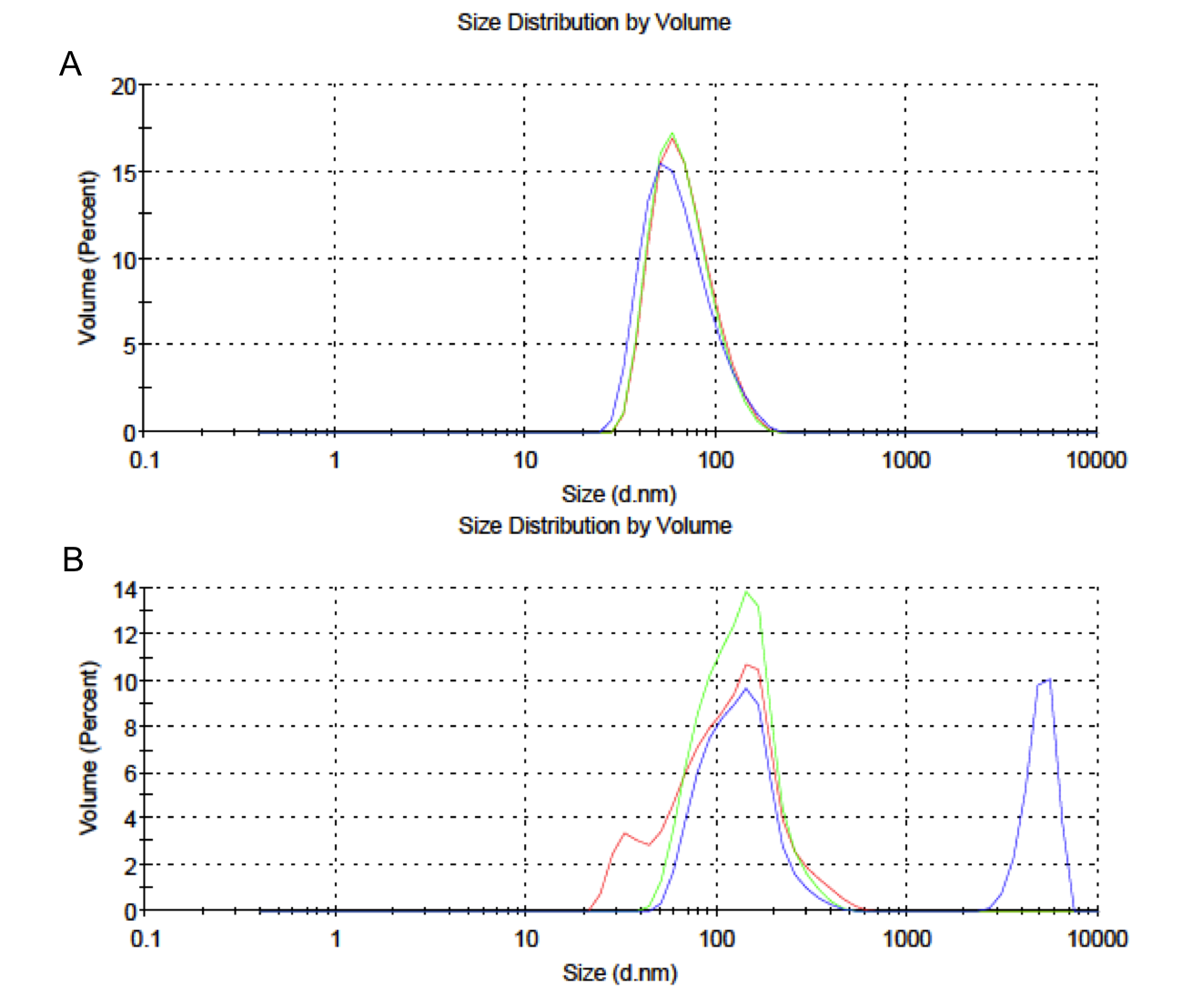


**Figure S3. Size distribution by volume measured by DLS.** (A) bare nanodiamonds and (B) funtionalized nanodiamonds.

**
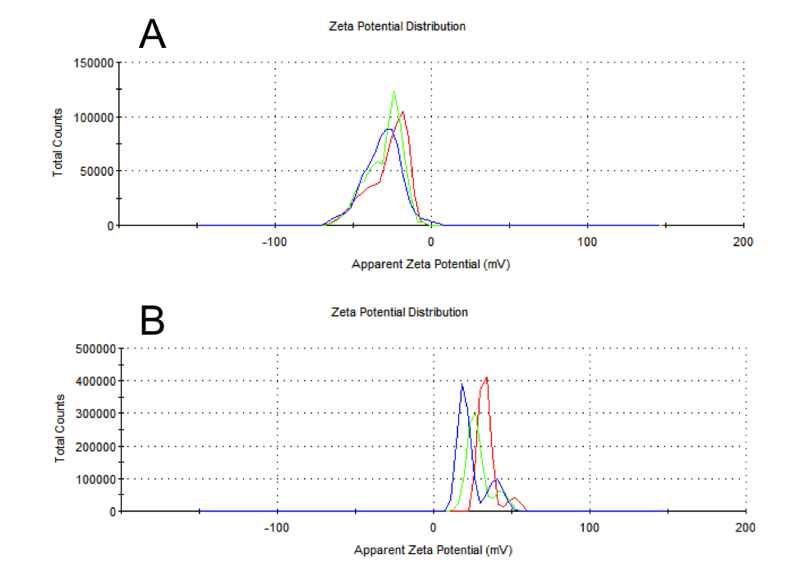
**

**Figure S4. Z- potential values.** (A) bare nanodiamonds and (B) funtionalized nanodiamonds.

Using atomic force microscopy (AFM) we further characterize the size of bare nanodiamonds. The original nanodiamond solution was diluted in distale water and were spincoated on a glass surface.


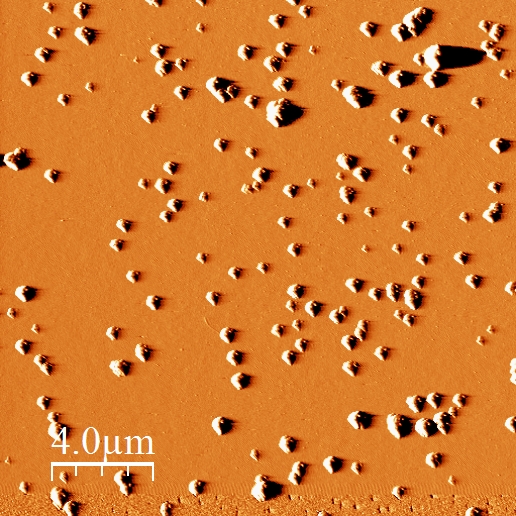


**Figure S5. AFM characterization oft he size of nanodiamonds.** Left: AFM image of non-functionalized nanodiamonds. Right: Histogram oft he size of nanodiamonds estimated from the AFM image.

**Initial molarity of nanodiamonds**

The concentration of the commercial sample is approximately 1 mg/ml and the mass of each nanodiamond is approximately 7 x 10^-17^ g, which is calculated as the volume of a 35 nm diameter spherical nanocrystal (2x10^-23^ m^3^) multiplied by the density of diamond (3.5x10^-6^ gr/m^3^). Therefore, the number of nanodiamonds per liter is 1.4x10^16^ . Therefore, dividing this number by Avogadro’s number, we obtain an initial molarity of 23x10^-9^ mol/L, or 23 nM. Starting from this concentration, the molarity of the different dilutions was calculated.

**Structure characterization of nanodiamonds**

Using high-resolution tunneling electron microscopy we characterized the structure of nanodiamonds.

**
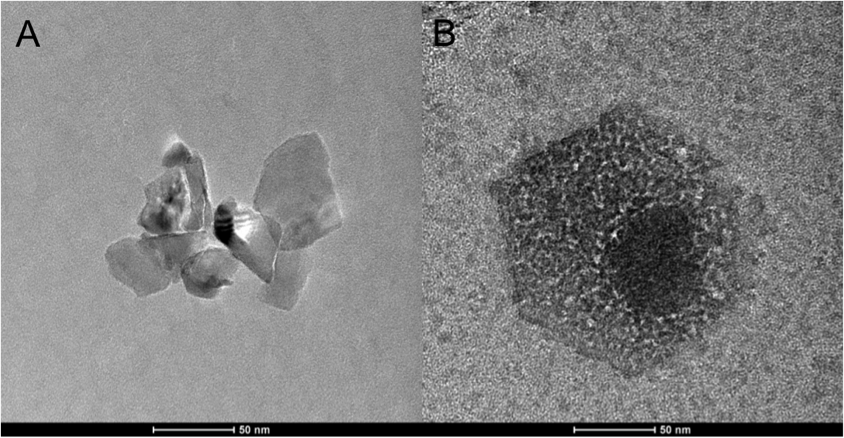
**

**Figure S6. Structure of nanodiamonds.** HR-TEM. Electron micrographs showing A) NDs and B) fNDs.

**Further experimental controls**

**
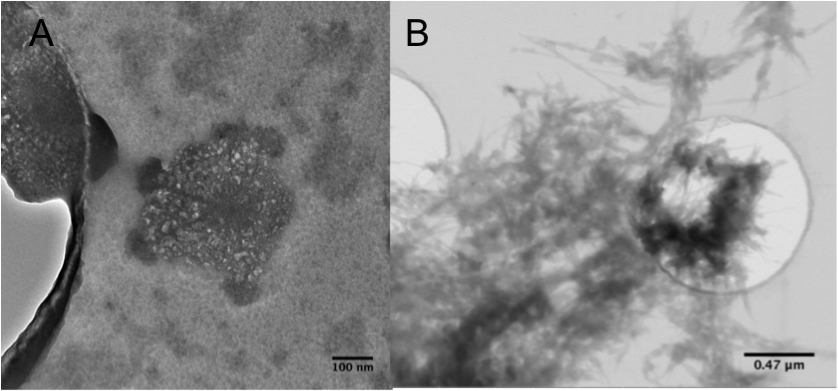
**

**Figure S7. Association of fNDs with albumin fibers. STEM Images.** fNDs were incubated with albumin fibers to evaluate an unspecific interaction of fNDs with another kinds of fibers. No interaction between albumin fibers and fNDs was observed. Free fNDs (a) and albumin fibers (b) were observed separately.

**
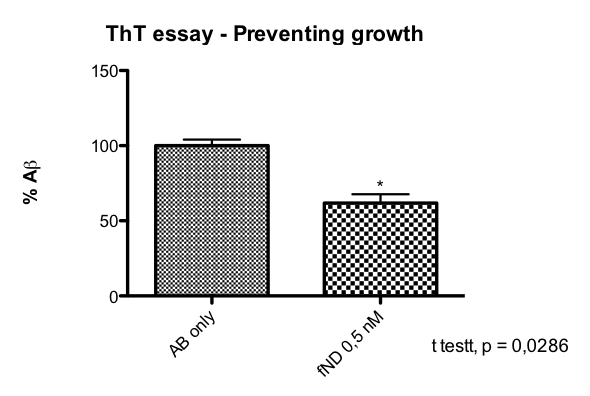
**

**Figure S8. Thioflavine-T results.** Fluorescence intensity signal from samples of Aβ-amyloid fibrils in presence of 0.5 nM of fND. The results are expressed as percentages with respect to the intensity from Aβ-amyloid fibrils free peptide.


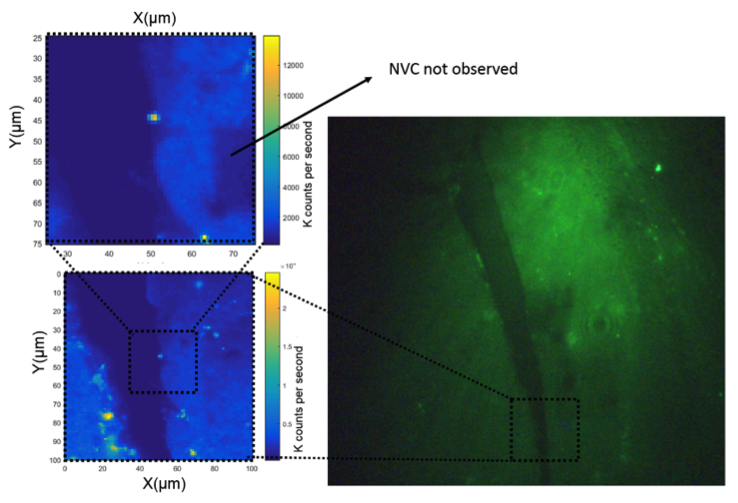


**Figure S9. Association of fNDs with Aβ fibers and plaques.** Composed confocal image of AD mouse brain tissue slides stained to detect Aβ plaques with an anti Aβ antibody 4G8 and Alexa 488 (green points) secondary antibody; and fNDs illuminated with 532 nm laser. Inset: zoom outside the neighborhood of Aβ plaque where we are not able to detect fNDs.

**
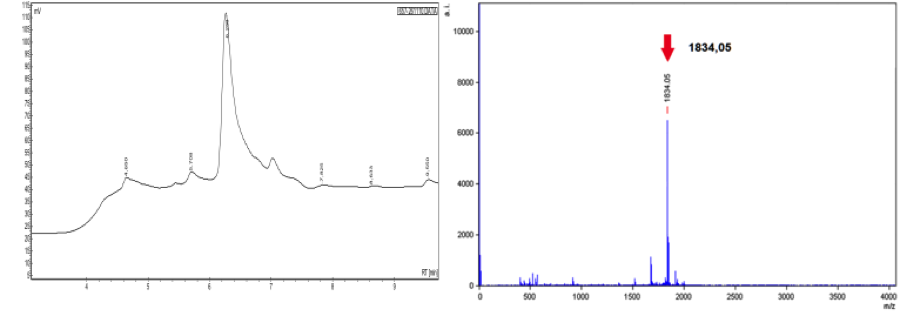
**

**Figure S10. HPLC chromatogram and MS/MALDI TOF spectrum of the R7CLPFFD.** The HPLC chromatogram was realized with a column Kromasil 100-5C18 (250 x 4.6 mm), using a gradient 0-40 % acetonitrile whit a retention time of 30 min and λ of detection was 200 nm. Mass spectra were performed using a matrix of 2,5- dihidrobenzóico acid (DHB) and α-cyano-4-hydroxycinnamic acid (ACH) at a concentration of 10 mg/mL in acetonitrile/formic acid 0.1% v/v (1:2).
